# Deep Eutectic Solvent-Assisted Extraction, Partially Structural Characterization, and Bioactivities of Acidic Polysaccharides from Lotus Leaves

**DOI:** 10.3390/foods10102330

**Published:** 2021-09-30

**Authors:** Ding-Tao Wu, Kang-Lin Feng, Ling Huang, Ren-You Gan, Yi-Chen Hu, Liang Zou

**Affiliations:** 1 Sichuan Engineering & Technology Research Center of Coarse Cereal Industralization, Key Laboratory of Coarse Cereal Processing (Ministry of Agriculture and Rural Affairs), School of Food and Biological Engineering, Chengdu University, Chengdu 610106, China; lhsicau184@163.com (L.H.); ganrenyou@caas.cn (R.-Y.G.); huyichen0323@126.com (Y.-C.H.); 2 Institute of Food Processing and Safety, College of Food Science, Sichuan Agricultural University, Ya’an 625014, China; klfsicau@163.com; 3 Research Center for Plants and Human Health, Institute of Urban Agriculture, Chinese Academy of Agricultural Sciences, Chengdu 610213, China

**Keywords:** lotus leaf, deep eutectic solvent assisted extraction, polysaccharide, chemical structure, biological activity

## Abstract

Lotus leaves are often discarded as byproducts in the lotus industry. Polysaccharides are regarded as one of the essentially bioactive components in lotus leaves. Therefore, in order to promote the application of lotus leaves in the functional food industry, the deep eutectic solvent (DES) assisted extraction of polysaccharides from lotus leaves (LLPs) was optimized, and structural and biological properties of LLPs extracted by DES and hot water were further investigated. At the optimal extraction conditions (water content of 61.0% in DES, extraction temperature of 92 °C, liquid-solid ratio of 31.0 mL/g and extraction time of 126 min), the maximum extraction yield (5.38%) was obtained. Furthermore, LLP-D extracted by DES and LLP-W extracted by hot water possessed the same sugar residues, such as 1,4-α-D-GalA*p*, 1,4-α-D-GalAMe*p*, 1,3,6-β-D-Gal*p*, 1,4-β-D-Gal*p*, 1,5-α-L-Ara*f*, and 1,2-α-L-Rha*p*, suggesting the presence of homogalacturonan (HG), rhamnogalacturonan I and arabinogalactan in both LLP-W and LLP-D. Notably, LLP-D was much richer in HG fraction than that of LLP-W, suggesting that the DES could assist to specifically extract HG from lotus leaves. Additionally, the lower molecular weight and higher content of uronic acids were observed in LLP-D, which might contribute to its much stronger in vitro antioxidant, hypoglycemic, and immunomodulatory effects. These findings suggest that the optimized DES assisted extraction method can be a potential approach for specific extraction of acidic polysaccharides with good bioactivities from lotus leaves for applications in the functional food industry.

## 1. Introduction

Lotus (*Nelumbo nucifera*), a common water plant of the family *Nelumbonaceae*, is widely cultivated in Asia [1,2]. Lotus has also been cultivated as an industrial crop in China, and is widely used as an edible and medicinal plant [1,2]. Usually, lotus is mainly composed of petal, seed, leaf, and root. Among them, lotus leaf is considered as one of the mainly edible parts of lotus, which is also used as an herbal medicine to treat diverse diseases, such as hyperlipidaemia, hypertension, obesity, and epistaxis [1,2]. A large number of studies have demonstrated that lotus leaves possess promising health-promoting effects, such as antioxidant, anti-obesity, hypoglycemic, hypolipidemic, anti-cancer, hepatoprotective, and anti-inflammatory effects [2]. Generally, these health benefits are associated with a variety of bioactive components that exist in lotus leaves, such as polysaccharides, phenolic compounds, alkaloids, and essential oils [2]. Among them, polysaccharides, as the essentially bioactive components in lotus leaves, also possess various health-promoting effects, such as anti-diabetic, antioxidant, antiglycation, and immunostimulatory effects [3,4,5,6,7]. Therefore, all data cited above indicates that polysaccharides extracted from lotus leaves exhibit good application potentials in the functional food industry. However, although the annual production of lotus leaves ranges from 7000 to 10,000 tons in China, most of them are often discarded as byproducts in the lotus industry, and only 1% of them are applied in the tea and catering industry [2]. Therefore, in order to promote the application of lotus leaves in the functional food industry, the development of green and efficient methods for the preparation of bioactive polysaccharides from lotus leaves is necessary.

Generally, the extraction yield, chemical structure, and bioactivity of natural polysaccharides are greatly influenced by extraction methods [8,9,10]. Therefore, the optimization of the extraction efficiency of polysaccharides from lotus leaves is important for their future research, development, and application in the food industry. Recently, hot water extraction [7], enzyme-assisted extraction [5], and dynamic high pressure microfluidization-assisted extraction (DHPMAE) [3] have been applied for extracting polysaccharides from lotus leaves. Indeed, different extraction techniques obviously affect the chemical and biological properties of polysaccharides extracted from lotus leaves. However, these methods possess some drawbacks of low extraction efficiency, high economic cost, and specific requirement of equipment. More recently, deep eutectic solvent (DES) has emerged to extract biopolymers and various plant compounds [11]. DES is a kind of new extracting solvent, which is composed of a hydrogen bonding acceptor and a hydrogen bonding donor [12]. As a green solvent, DES has advantages of low economic cost, safety, biodegradation, and high dissolution ability [13]. DES can be served as extracting solvent for multiple bioactive components, such as flavonoids, phenolic acids, and alkaloids [12]. Notably, DES has also been utilized to extract various polysaccharides from plants, which has been proven to exhibit higher extraction yield than that of traditional extraction solvent [14,15,16]. Therefore, all data suggests that DES possesses good potential for efficient extraction of polysaccharides from edible and medicinal plants.

However, up to now, to the best of our knowledge, the potential applicability of DES for extracting bioactive polysaccharides from lotus leaves (LLPs) has never been investigated. Besides, the possible effect of DES assisted extraction on chemical structures and bioactivities of LLPs was still unknown. Hence, the objective of this study was to explore the potential applicability of DES for efficient extraction of LLPs by using Box-Behnken Design. Subsequently, the chemical structures and bioactivities of LLPs prepared by DES assisted extraction were investigated, and compared with that of hot water extraction.

## 2. Materials and Methods

### 2.1. Materials and Reagents

Lotus leaves (*Nelumbo nucifera* Gaertn) were collected from Meishan, Sichuan Province, China. Lotus leaves were dried by hot wind at 75 °C for 7 h according to a previous study [6], and then pulverized into powder.

Choline chloride, ethylene glycol, 2-diphenyl-1-picrylhydrazyl (DPPH), 3-ethylbenzthiazoline-6-sulphonic acid (ABTS), hydroxytoluene (BHT), 4-nitrophenyl β-D-glucopyranoside (*p*NPG), α-glucosidase (10 U/mg), α-amylase (1000 U/mg), acarbose, 1-phenyl-3-methyl-5-pyrazolone (PMP), vitamin C, and soluble starch were purchased from Merck (Merck Ltd., Darmstadt, Germany).

### 2.2. Extraction of Polysaccharides from Lotus Leaves (LLPs)

#### 2.2.1. Preparation of Raw Material

Lotus leaves (5.0 g) were mixed with ethanol (50.0 mL, 80%, *v*/*v*) at room temperature for 2.0 h to remove ethanol-soluble constituents by using an ultrasonic cleaner (Ningbo Scientz Biotechnology Co., Ltd., Ningbo, China). After centrifugation (5000× *g*, 10 min), the extracted residues were collected and applied for the further preparation of polysaccharides.

#### 2.2.2. Hot Water Extraction of LLPs

Hot water extraction (HWE) was performed based on our previous study [10]. Briefly, 5.0 g of extracted residues of lotus leaves were mixed with 150.0 mL of deionized water, and stirred at heating magnetic stirrer at 90 °C for 3.0 h. After centrifugation (4000× *g*, 20 min), the supernatant was precipitated with four volumes of 95% (*v*/*v*) ethanol and placed in 4 °C for 12 h. Precipitations were obtained by centrifugation (4000× *g*, 15 min), and washed with ethanol (76%, *v*/*v*). Then, precipitations were redissolved and further dialyzed (molar mass cut off, 3.5 kDa) against deionized water to remove small molecules. Finally, polysaccharides from lotus leaves extracted by HWE (LLP-W) were obtained by freeze drying.

#### 2.2.3. Deep Eutectic Solvent Assisted Extraction (DESE) of LLPs

DES was prepared by using choline chloride (ChCl) and ethylene glycol (EG) in a molar ratio of 1:3 according to previous studies with slight modifications [17,18]. Then, the mixture was stirred at 80 °C until the solution became clear and stable to obtain a DES stocking solution.

For the DESE extraction, the single factor experiments were firstly used to investigate effects of content of water in DES (30, 40, 50, 60, and 70%), extraction temperature (60, 70, 80, 90, and 100 °C), liquid-solid ratio (20, 30, 40, 50, and 60 mL/g), and extraction time (60, 90, 120, 150, and 180 min) on the yields of LLPs. Each single factor was optimized when other factors were fixed. Briefly, 5.0 g of extracted residues were mixed with DES extraction solvent, and extracted on the heating magnetic stirrer. Then, the following steps were conducted in accord with Section 2.2.2 to obtain lotus leaves polysaccharides extracted by DESE (LLP-D).

Furthermore, according to the results from the single factor experiments, a three-level Box-Behnken design (BBD) with four-factors was used to optimize the DESE conditions. The independent variables were content of water in DES (X_1_, 50, 60, 70%), extraction temperature (X_2_, 80, 90, 100 °C), liquid-solid ratio (X_3_, 20, 30, 40, mL/g), and time (X_4_, 90, 120, 150 min). Table 1 shows the independent variables and their levels. Each experiment was tested in triplicate. Then, the experimental data of BBD was utilized for modeling and optimizing the experimental conditions by using response surface methodology (RSM). The experimental data for the response was fitted by using the second-order polynomial model and the equation was as follow:

(1) $${Y=\beta }_{0}+\overset{3}{\underset{i=1}{\sum }}\beta_{i}X_{i}+\overset{3}{\underset{i=1}{\sum }}\beta_{\mathrm{ii}}X_{i}^{2}+\overset{2}{\underset{i=1}{\sum }}\overset{3}{\underset{j=i+1}{\sum }}\beta_{\mathrm{ij}}X_{i}X_{j}$$

where Y is the predicted response; X_i_ and X_j_ are different variables; and β_0_, β_i_, β_ii_, and β_ij_ are the regression coefficients for intercept, linearity, square, and interaction, respectively [16].

### 2.3. Physicochemical and Structural Characterization of Polysaccharides from Lotus Leaves (LLPs)

The contents of total polysaccharides, proteins, and uronic acids in LLP-D and LLP-W were analyzed by previously reported colorimetric methods [19]. The molecular weights and molecular weight distributions of LLPs were investigated by using high performance size exclusion chromatography equipped with the multi angle laser light scattering and the refractive index detector (Wyatt Technology Co., Santa Barbara, CA, USA) [20]. Besides, the apparent viscosities of LLPs were determined at the concentration of 10.0 mg/mL by using a Discovery Hybrid Rheometer-1 (TA instruments, New Castle, DE, USA) with a parallel steel plate (40.0 mm diameter and 1.0 mm gap) [20]. The constituent monosaccharides of LLPs were analyzed by using Thermo U3000 HPLC system (Thermo Fisher Scientific, Waltham, MA, USA) equipped with a Phenomenex gemini 5 μ C18 (150 mm × 4.6 mm) column followed by a formerly reported method [20]. Moreover, the FT-IR spectra of LLPs were recorded to analyze their chemical structures by using a Nicolet iS 10 FT-IR (ThermoFisher scientific, Waltham, MA, USA) [20]. Furthermore, the ^1^H and ^13^C NMR spectra of LLPs were also recorded to further analyze their chemical structures by using a Bruker Ascend 600 MHz spectrometer with a z-gradient probe (Bruker, Rheinstetten, Germany) [20].

### 2.4. Evaluation of Bioactivities of Polysaccharides from Lotus Leaves (LLPs)

#### 2.4.1. Evaluation of In Vitro Antioxidant Activities of LLPs

The ABTS, DPPH, and NO radical scavenging capacities as well as ferric reducing antioxidant power (FRAP) of LLPs were detected by previously reported methods [10]. LLP-D and LLP-W were detected at five different concentrations against ultrapure water as the blank control, and the BHT (for DPPH assay) or vitamin C (for ABTS, NO and FRAP assays) was applied as the positive control.

#### 2.4.2. Evaluation of In Vitro Hypoglycemic Effects of LLPs

α-Amylase and α-glucosidase inhibitory effects of LLPs were also detected by formerly reported methods [20]. LLPs were tested at five different concentrations with acarbose as the positive control. Then, IC_50_ values (µg/mL) of LLPs were calculated through a log-regression curve.

#### 2.4.3. Evaluation of Immunomodulatory Activities of LLPs

The immunomodulatory effects of LLPs were also evaluated according to the formerly reported method [20]. Briefly, RAW 264.7 macrophages were cultivated in an anaerobic incubator with the RPMI-1640 medium at 37 °C. The cells (1 × 10^5^/well) were treated with LLPs at the concentrations ranged from 5 to 320 μg/mL, and cultured for 24 h. The cultural medium and lipopolysaccharide (LPS, 1.0 μg/mL) were used as blank and positive controls, respectively. Effects of LLP-W and LLP-D on the cell viability of RAW 264.7 macrophages were measured by using a MTT colorimetric method. In addition, the nitric oxide (NO) production of RAW 264.7 macrophages was determined by Griess reagent. Furthermore, the interleukin-6 (IL-6) and tumor necrosis factor-alpha (TNF-α) secretion of RAW 264.7 macrophages after incubated with LLP-D and LLP-W were determined by an ELISA kit (eBioscience, San Diego, CA, USA).

### 2.5. Statistical Analysis

Each experiment was operated in triplicate, and the data were displayed as the means ± standard deviations. The experimental design, modeling, and data analysis were performed by Design Expert software 8.0.6.1 (Stat-Ease Inc., Minneapolis, MN, USA). Statistical analysis was conducted using Origin 9.0 software (OriginLab Corporation, Northampton, MA, USA).

## 3. Results and Discussion

### 3.1. Optimization of DES Assisted Extraction of LLPs

#### 3.1.1. Single Factor Experimental Analysis

**Figure 1:**
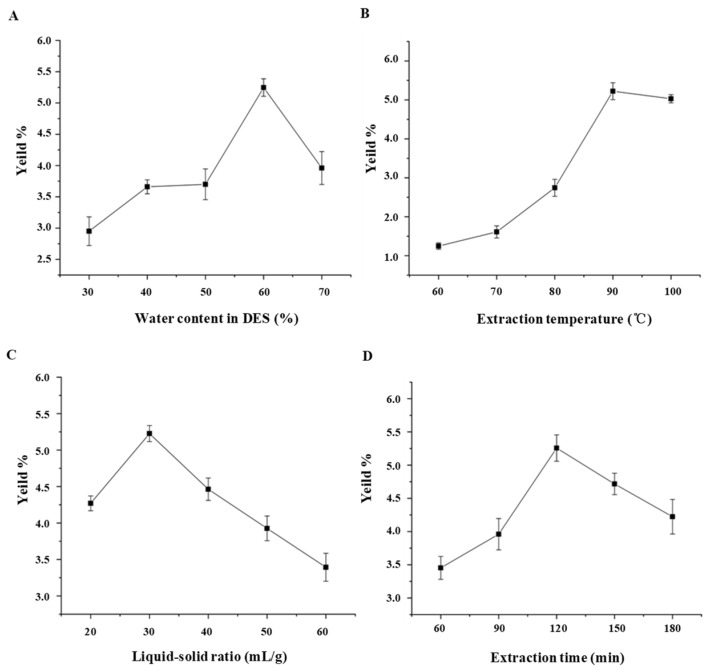
Effects of water content (**A**) in deep eutectic solvent (DES), extraction temperature (**B**), liquid-solid ratio (**C**), and extraction time (**D**) on extraction yields of polysaccharides extracted from lotus leaves.

Recent studies have demonstrated that the DES ChCl-EG is much suitable for the extraction of polysaccharides from natural resources [17,18]. Therefore, the DES ChCl-EG was chosen as the extraction solvent for the following studies. Figure 1 shows the effects of water content in DES, extraction temperature, liquid-solid ratio, and extraction time on the extraction yields of LLPs. Generally, the content of water in DES can change its viscosity and polarity, resulting in a difference on the extraction yield of polysaccharides [16]. The highest extraction yield of LLPs was obtained at the water content of 60% (*v*/*v*) in DES. This result may be due to that the higher viscosity of DES extraction solvent caused by the lower water content makes it difficult to penetrate into the plant cell to extract polysaccharides efficiently, while an excess of water content in DES may limit the interactions between polysaccharides and DES [14,16]. The extraction temperature was another important parameter for polysaccharide extraction. The extraction yield of LLPs gradually increased with the increase of extraction temperature from 60 °C to 90 °C, and slightly decreased at the temperature of 100 °C. This phenomenon suggested that a relatively high extraction temperature could reduce the transfer resistance, decrease the viscosity of extraction solvent, and increase the solubility of LLPs [16,21]. However, an excessively high extraction temperature may cause the degradation of LLPs to decrease the extraction yield [16,21]. Besides, the liquid-solid ratio also has an influence on the yield of LLPs [15]. The extraction yield of LLPs increased from 20 mL/g to 30 mL/g, and decreased above 30 mL/g. Furthermore, the extraction yield of LLPs increased with the increase of extraction time from 60 to 120 min, while a decreasing was found after 120 min. This result might be caused by the hydrolysis of LLPs under a long extraction time [21]. Finally, the optimal extraction conditions of single factor experiment were measured as follows, 60% of water content in DES, temperature of 90 °C, liquid-solid ratio of 30 mL/g, and time of 120 min.

#### 3.1.2. Box-Behnken Design Analysis

A Box-Behnken design (BBD) with 29 runs was further used to optimize the extraction conditions of DESE. Based on the BBD matrix and experimental results in Table 1, the predicted mathematical model was expressed by the following second-order polynomial equation through multiple regression analysis,

(2) $$Y=5.36\;+\;{0.19X}_{1}+{0.43X}_{2}+{0.18X}_{3}+{0.27X}_{4}-{0.19X}_{1}X_{2}+{0.1X}_{1}X_{3}-\;{0.005X}_{1}X_{4}\;-{0.027X}_{2}X_{3}+{0.2X}_{2}X_{4}-{0.067X}_{3}X_{4}-{1.18X}_{1}^{2}-{1.02X}_{2}^{2}-{0.68X}_{3}^{2}-{0.8X}_{4}^{2}$$

where Y is the yield of LLPs, X_1_, X_2_, X_3_ and X_4_ are the content of water in DES (%), extraction temperature (°C), liquid-solid ratio (mL/g), and extraction time (min), respectively.

The influence of the variables on the extraction yield of LLPs and the validity of the fitted model were evaluated by analysis of variance (ANOVA). As shown in Table 2, the extremely low *p*-value (*p* < 0.0001) and the high *F*-value (147.70) suggested that the regression model was statistically significant [14]. Indeed, the *F*-value (3.06) and *p*-value (0.1464) of the lack of fit verified the validity of the regression model [14]. The coefficient of determination (R^2^) was 0.9933, which was very close to the value of adjusted coefficient determination (R^2^_adj_ = 0.9866), suggesting that there was a high degree of correlation between the predicted yield and the actual yield of LLPs [3,14]. Besides, the values of the coefficient of variation and the adequate precision were 2.53% and 43.16, respectively, indicating that the fitting model possessed good precision, reliability, and reproducibility [3,16]. Furthermore, the linear coefficients (X_1_, X_2_, X_3_, X_4_), interaction coefficients (X_1_X_2_, X_1_X_3_, X_2_X_4_), and quadratic term coefficients (X_1_^2^, X_2_^2^, X_3_^2^, X_4_^2^) of the fitted model equation were significant with *p*-values lower than 0.05. Results indicated that the selected extraction parameters, including content of water in DES, extraction temperature, liquid-solid ratio, and extraction time, were the determinant parameters for the polysaccharide extraction process.

**Figure 2:**
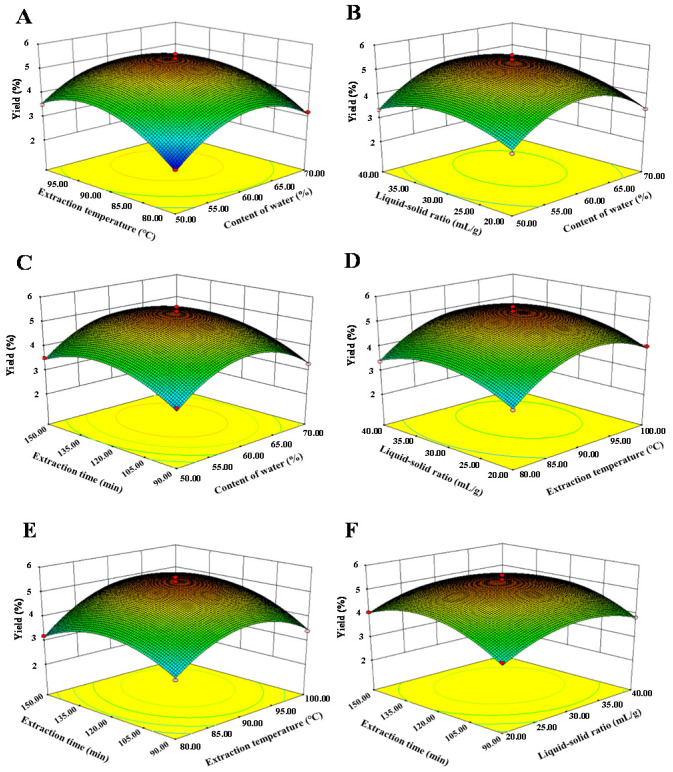
Three-dimensional response surface plots of deep eutectic solvent assisted extraction. (**A**) interaction between content of water and extraction temperature; (**B**) interaction between content of water and liquid-solid ratio; (**C**) interaction between content of water and extraction time; (**D**) interaction between liquid-solid ratio and extraction temperature; (**E**) interaction between extraction time and extraction temperature; (**F**) interaction between extraction time and liquid-solid ratio.

**Figure 3:**
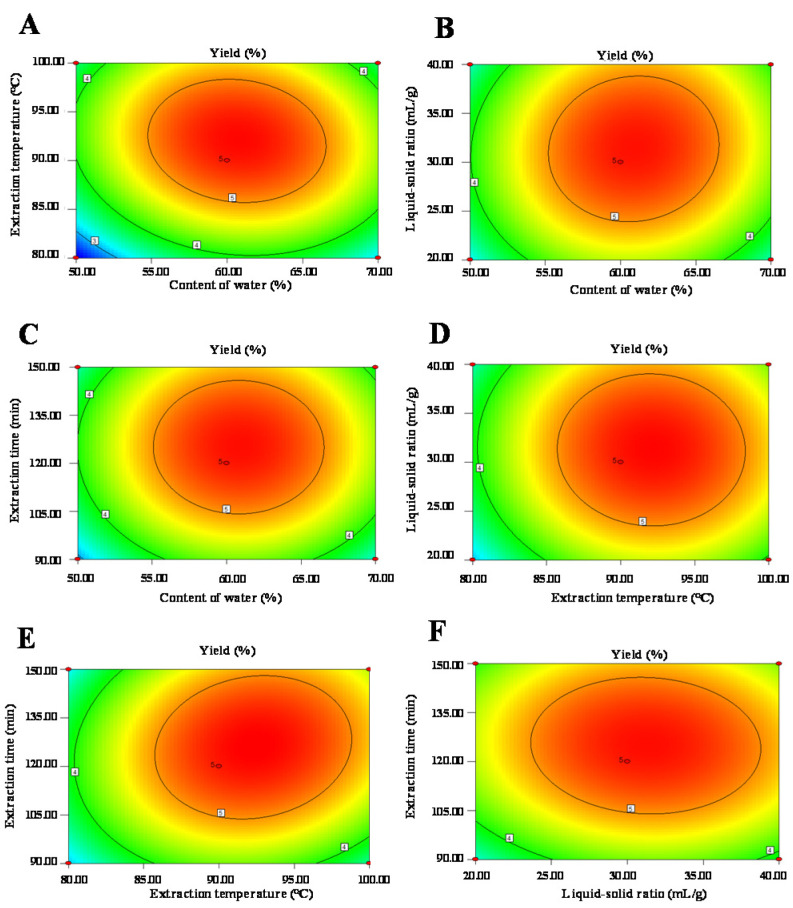
Two-dimensional contour plots of deep eutectic solvent assisted extraction. (**A**) interaction between content of water and extraction temperature; (**B**) interaction between content of water and liquid-solid ratio; (**C**) interaction between content of water and extraction time; (**D**) interaction between liquid-solid ratio and extraction temperature; (**E**) interaction between extraction time and extraction temperature; (**F**) interaction between extraction time and liquid-solid ratio.

Furthermore, Figure 2 and Figure 3 show the 3D response surface plots and the 2D contour plots of the regression equation. It could be estimated that the content of water in DES and the extraction temperature had more positive effects on the extraction yield of LLPs than that of the extraction time and the liquid-solid ratio, respectively. Generally, the shape of the contour plot is elliptical, suggesting that the mutual interaction between the two extraction parameters is notable [21]. While shape of the contour plot is circular, indicating that the mutual interaction between the two extraction parameters is not significant [21]. As shown in Figure 3, significant interaction effects between the content of water in DES and the extraction temperature, the extraction temperature and the extraction time, and the content of water in DES and the liquid-solid ratio were observed visually [21], which were the same as shown in Table 2. Moreover, according to the analysis of the regression model equation, it was concluded that the optimal extraction conditions were as follows: 60.69% of water content in DES, extraction temperature of 92.19 °C, liquid-solid ratio of 31.22 mL/g, and extraction time of 125.73 min. Considering the practical feasibility, the operating conditions were adjusted as 61.0% of water content in DES, extraction temperature of 92.0 °C, liquid-solid ratio of 31.0 mL/g, and extraction time of 126.0 min. Under these optimal conditions, the actual extraction yield of LLPs was 5.38% ± 0.11% (*n* = 3), which is very close to the predicted value (5.45%).

### 3.2. Physicochemical and Structural Properties of LLPs

#### 3.2.1. Chemical Compositions of LLPs

The extraction yields and chemical compositions of LLP-D and LLP-W are expressed in Table 3. Results indicated that the extraction yield of LLP-D extracted by DES (5.38% ± 0.11%) was obviously higher than that of hot water extraction in the present study (3.22% ± 0.16%) and in the previous study (1.18%) [7]. Indeed, the extraction yield of LLP-D was also notably higher than that of enzyme assisted extraction (1.93%) [5]. Although the extraction yield of LLP-D is slightly lower than that of DHPMAE (6.31%) [3], the DHPMAE approach requires specific equipments with high costs, which limits its application in the food industry. Therefore, the developed DES assisted extraction method could be applied for efficient extraction of LLPs. Furthermore, both LLP-D extracted by DES (82.10% ± 1.29%) and LLP-W (77.94% ± 0.48%) extracted by hot water possessed high contents of total polysaccharides, indicating that polysaccharides were the major components in LLPs. Obviously, LLP-D (39.96% ± 0.32%) had significantly higher content of total uronic acids than that of LLP-W (22.98% ± 1.15%), indicating that the DES extraction solvent could aid to specifically extract acidic polysaccharides from natural resources [11]. In addition, it is reported that the contents of uronic acids have a great influence on the bioactivities of acidic polysaccharides [22]. Furthermore, minor proteins were determined in both LLP-D and LLP-W, suggesting that minor polysaccharide-protein complexes might exist in LLPs.

#### 3.2.2. Molecular Weights and Apparent Viscosities of LLPs

**Figure 4:**
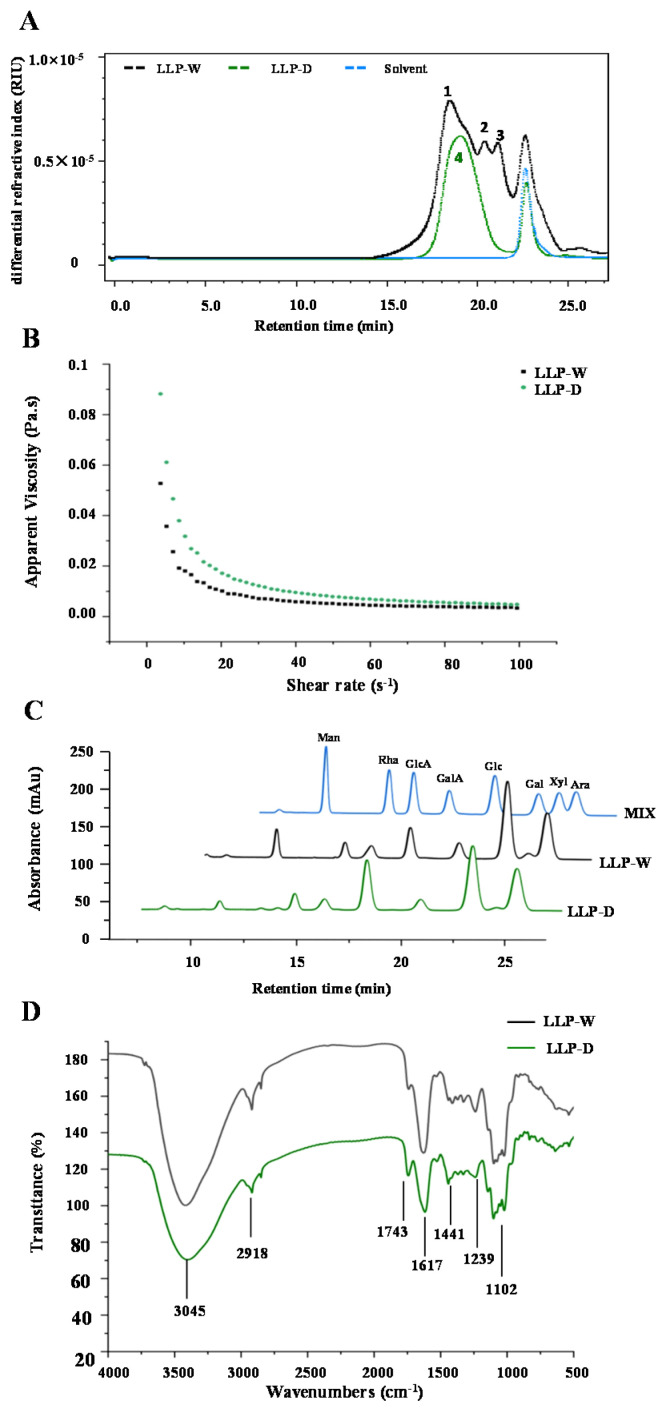
Size exclusion chromatograms (**A**), apparent viscosities on the shear rate (**B**), high performance liquid profiles of monosaccharides (**C**), and FT-IR spectra (**D**) of LLP-D and LLP-W. LLP-D and LLP-W are polysaccharides from lotus leaves extracted by deep eutectic solvent and hot water, respectively; Rha, rhamnose; GalA, galacturonic acid; Ara, arabinose; Gal, galactose; GlcA, glucuronic acid; Glc, glucose; Xyl, xylose.

Molecular weight and apparent viscosity have great influences on the bioactivities of polysaccharides. Hence, the molecular weights and apparent viscosities of LLP-D and LLP-W were investigated and compared. As displayed in Figure 4A, the HPSEC-RID chromatograms of LLP-W and LLP-D were notably different. Three polysaccharide fractions (fractions 1 to 3) were found in LLP-W, in accordance with the previous study that several polysaccharide fractions exist in the lotus leaves polysaccharides extracted by hot water [5]. Interestingly, only one single and symmetric polysaccharide fraction (fraction 4) was detected in LLP-D, suggesting that the DESE method could be helpful for specific extraction of polysaccharides from lotus leaves with a high content of uronic acids (Table 3). Furthermore, molecular weights of different polysaccharide fractions existed in LLP-W were measured to be 12.90 × 10^4^ Da (fraction 1), 4.70 × 10^4^ Da (fraction 2), and 3.43 × 10^4^ Da (fraction 3), respectively. Compared with LLP-W, a lower molecular weight (4.03 × 10^4^ Da) of polysaccharide fraction 4 in LLP-D was determined. Usually, polysaccharides with lower molecular weights exhibit higher antioxidant capacities against free radicals [22,23].

Moreover, apparent viscosities of LLP-D and LLP-W associated with the shear rates were investigated. As shown in Figure 4B, the apparent viscosities of LLP-W and LLP-D were decreased as the increase of shearing rate, conforming to a shear-thinning behavior. This phenomenon could be explained by the decrease of entanglement degrees among molecular chains with the increase of shear rate. In addition, LLP-D exhibit higher apparent viscosities than that of LLP-W. Usually, several factors, such as molecular weight, monosaccharide composition, degree of esterification, could obviously influence the apparent viscosities of natural polysaccharides [24]. In this study, the relatively high apparent viscosity of LLP-D might be attributed to its high content of uronic acids and high degree of esterification (Table 3).

#### 3.2.3. Monosaccharide Compositions of LLPs

HPLC followed by PMP derivatization was used to detect the monosaccharide compositions of LLPs. Figure 4C shows the HPLC profiles of monosaccharide compositions of LLPs. As shown in Figure 4C, LLP-W and LLP-D had the same types of monosaccharides, which were composed of galactose (Gal), arabinose (Ara), galacturonic acid (GalA), rhamnose (Rha), mannose (Man), glucose (Glc), glucuronic acid (GlcA), and xylose (Xyl). Table 3 summarizes the molar ratios of Rha, Gal, Ara, GalA, Man, Glc, and GlcA in LLPs, which suggested that the major monosaccharides in LLPs extracted by DES and hot water were similar, including Gal, GalA, and Ara. Generally, the typical constituent monosaccharides for homogalacturonan (HG) and rhamnogalacturonan I (RG I) pectic domains are GalA, GlcA, Gal, Rha, and Ara while for hemicelluloses they are Man, Glc, and Xyl [11]. In addition, Ara and Gal can also arise from arabinogalactan (AG) [11]. Therefore, a large amount of HG and arabinogalactan as well as a few of RG I might exist in both LLP-W and LLP-D according to the molar ratios of their compositional monosaccharides. Indeed, the HG pectic domain was much higher in LLP-D than that of LLP-W according to the molar ratios of GalA and Rha, which further confirmed that acidic polysaccharides from lotus leaves were more easily to be extracted by DES than that of hot water. All abovementioned results suggested that the DES ChCl-EG extraction solvent had good potential for the extraction of acidic polysaccharides from lotus leaves.

#### 3.2.4. FT-IR and NMR Spectra of LLPs

Both FT-IR spectra and ^1^H and ^13^C NMR spectra were applied to analyze the structural features of LLP-D and LLP-W. As displayed in Figure 4D, LLP-W and LLP-D exhibited similar FT-IR spectra, indicating that LLP-W and LLP-D possessed similar functional groups. Specifically, the strong absorption bands at 3045 cm^−1^ and 2918 cm^−1^ were attributed to the stretching vibrations of O-H and C-H groups [5]. The absorption bands at 1743 cm^−1^ and 1617 cm^−1^ were attributed to the C=O vibrations of esterified carboxylic groups and free carboxylic groups, respectively, demonstrating that both LLP-W and LLP-D were acidic polysaccharides [8]. The absorption band at 1417 cm^−1^ further affirmed the presence of uronic acids [9]. Besides, the absorption bands at 1239 cm^−1^ and 1102 cm^−1^ were related to the C-O-C stretching vibration and the presence of pyranose sugars in LLPs, respectively [3,9]. Furthermore, the intensity of absorption band of 1743 cm^−1^ which associated with the carboxylic ester was higher in LLP-D than that of LLP-W, indicating that the degree of esterification (DE) of LLPs was obviously affected by the DES assisted extraction. Indeed, the DE values of LLP-D and LLP-W were determined to be 28.92% ± 0.77% and 16.46% ± 0.08%, respectively.

**Figure 5:**
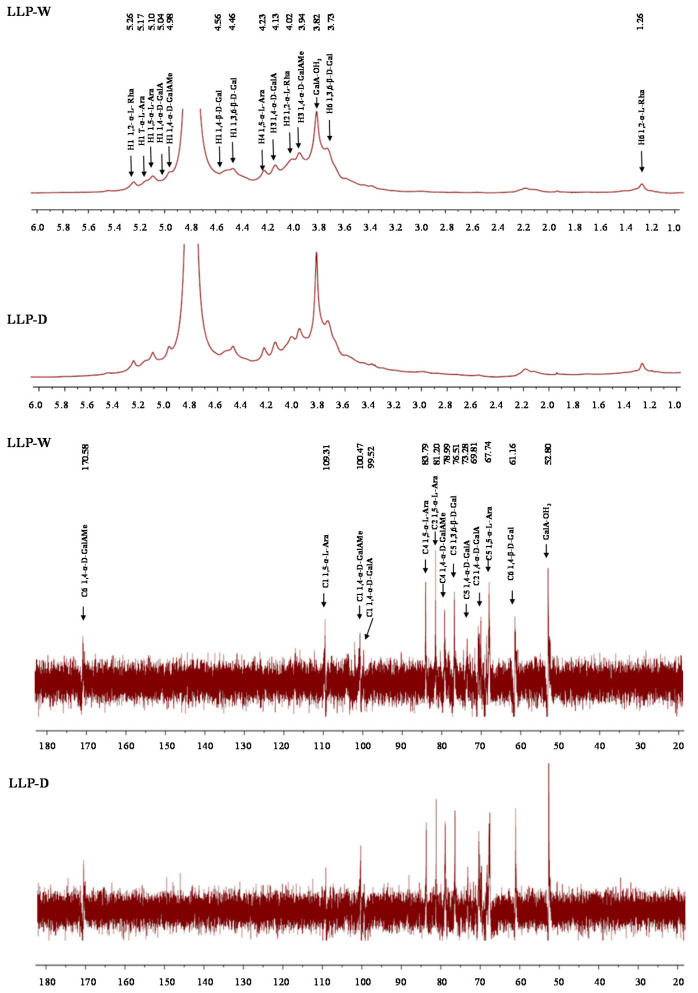
^1^H and ^13^C NMR spectra of LLP-D and LLP-W. LLP-D and LLP-W are polysaccharides from lotus leaves extracted by deep eutectic solvent and hot water, respectively.

Furthermore, Figure 5 shows the ^1^H and ^13^C NMR spectra of LLP-D and LLP-W. As shown in Figure 5, LLP-D and LLP-W exhibited similarly characteristic signals in both ^1^H and ^13^C NMR spectra, indicating that their primary structures were similar. The DES assisted extraction did not change the primary structure of acidic polysaccharides from lotus leaves. Specifically, the signal at 4.80 ppm was assigned to HDO. The signals at 5.26, 4.02, and 1.26 ppm were assigned to H-1, H-2, and H-6 of 1,2-α-L-Rha*p* [20,25,26]. The signals at 5.10/109.31 ppm and 4.23/83.79 ppm indicated the presence of H-1/C-1 and H-4/C-4 of 1,5-α-L-Ara*f*, whereas the C-2 and C-5 of 1,5-α-L-Ara*f* were measured to be 81.20 and 67.74 ppm [25,27]. Besides, the signal at 5.17 ppm was attributed to the H-1 of T-α-L-Ara*f* [25,28]. The peaks at 5.04/99.52 ppm were attributed to H-1/C-1 of 1,4-α-D-GalA*p*, whereas H-3, C-2, and C-5 of 1,4-α-D-GalA*p* were also determined to be 4.13, 69.81, and 73.28 ppm [20,25]. In addition, peaks at 4.98/100.47 were attributed to H-1/C-1 of 1,4-α-D-GalAMe*p*, whereas the H/C signals of the methyl signal of 1,4-α-D-GalAMe*p* were measured at 3.82 and 52.80 ppm [20,25]. Indeed, the signal at 170.58 ppm was assigned to the C-6 of 1,4-α-D-GalAMe*p* [25]. Compared with LLP-W, the intensities of peaks at 4.98 and 3.82 ppm of LLP-D were much stronger, which was similar with the FT-IR spectra that the DE value of LLP-D was higher than that of LLP-W (Table 3). Moreover, the signals at 4.46, 3.73, and 76.51 ppm were assigned to the H-1, H-6, and C-5 of 1,3,6-β-D-Gal*p* [29], and the signals at 4.56 and 61.16 ppm were attributed to the H-1 and C-6 of 1,4-β-D-Gal*p* [20,28]. Overall, according to the monosaccharide compositions, FT-IR spectra, and NMR spectra of LLPs, the main polysaccharide fractions, including HG, AG, and RG I, existed in LLPs [20,27,30].

### 3.3. In Vitro Antioxidant Activities of LLPs

**Figure 6:**
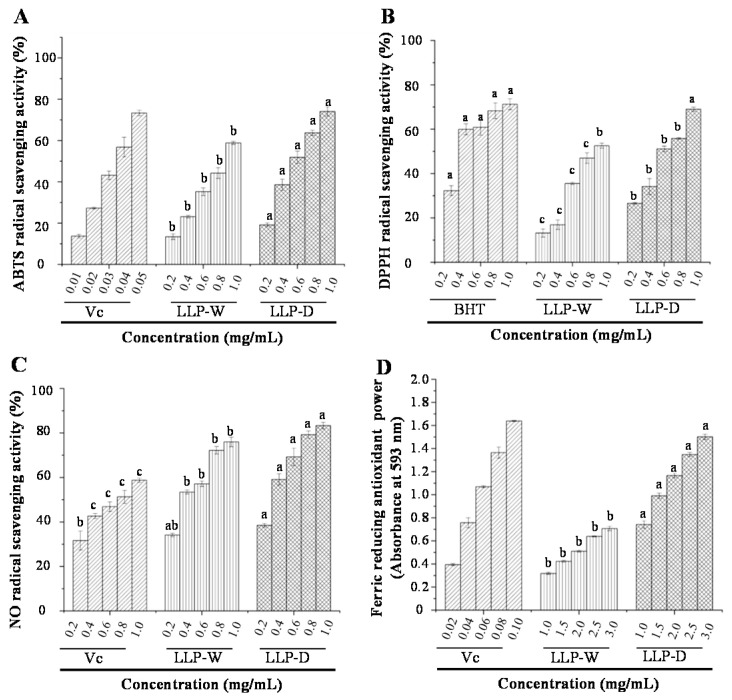
ABTS (**A**), DPPH (**B**), and NO radical scavenging activities (**C**), ferric reducing antioxidant powers (**D**). LLP-D and LLP-W are polysaccharides from lotus leaves extracted by deep eutectic solvent and hot water, respectively; Vc, vitamin C; BHT, butylated hydroxytoluene; Each experiment was operated in triplicate, and the error bars are standard deviations; Significant (*p* < 0.05) differences are shown by data bearing different letters (a–c).

In this study, ABTS, DPPH, NO radical scavenging capacities and ferric reducing antioxidant power (FRAP) of LLP-W and LLP-D were detected and compared. As shown in Figure 6A–D, both LLP-W and LLP-D exhibited remarkable antioxidant activities when compared with the positive controls (Vc and BHT). IC_50_ values of various different radical scavenging capacities of LLP-D and LLP-W were measured to be 0.61 ± 0.02 and 0.86 ± 0.01 mg/mL (ABTS), 0.65 ± 0.01 and 0.90 ± 0.01 mg/mL (DPPH), and 0.31 ± 0.03 and 0.43 ± 0.01 mg/mL (NO), respectively. Besides, the obvious antioxidant activities of LLP-D and LLP-W have also been demonstrated by the notable FRAP at the concentration of 3.0 mg/mL. Obviously, LLP-D exhibited significantly (*p* < 0.05) stronger antioxidant activities than that of LLP-W.

Generally, physicochemical properties of acidic polysaccharides, including molecular weight, type of monosaccharide, content of uronic acid, and degree of esterification, exhibit notable influences on their in vitro antioxidant abilities [20,22]. Specifically, the acidic polysaccharide with low molecular weight often exhibits strong antioxidant capacities, because it is easy to react with free radicals for its great proportion of exposed reducing ends and the large surface area [22,23]. Besides, the interaction between free uronic acid groups and carbon atoms can also contribute to the high antioxidant capacities of acidic polysaccharides [22,23]. Hence, compared with LLP-W, the stronger antioxidant capacities of LLP-D might be due to its lower molecular weight and higher content of free uronic acids (Table 3). These results indicated that the optimized DESE method had good potential for the extraction of acidic polysaccharides with remarkable antioxidant capacities from lotus leaves, which is beneficial to improve the application of lotus leaves in the food industry.

### 3.4. In Vitro Hypoglycemic Effects of LLPs

**Figure 7:**
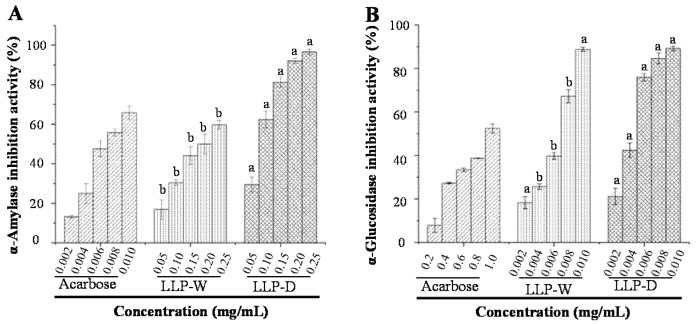
Inhibitory effects against α-amylase (**A**) and α-glucosidase (**B**) of LLP-D and LLP-W. Each experiment was operated in triplicate, and the error bars are standard deviations; Significant (*p* < 0.05) differences are shown by data bearing different letters (a and b).

The in vitro hypoglycemic activity of natural polysaccharides can be efficiently evaluated through the determination of their inhibitory rates on the activities of α-amylase and α-glucosidase [20,31,32]. Therefore, inhibitory effects of LLP-D and LLP-W against α-amylase and α-glucosidase were investigated. As shown in Figure 7A,B, both LLP-D and LLP-W exhibited potent inhibitory effects against α-amylase and α-glucosidase. Notably, LLP-D exhibited stronger inhibitory effects on α-amylase and α-glucosidase than that of LLP-W. The IC_50_ values of acarbose, LLP-D, and LLP-W against α-amylase were measured to be 7.32 ± 0.20, 80.02 ± 0.95, and 198.39 ± 12.07 μg/mL, whereas IC_50_ values of acarbose, LLP-D, and LLP-W against α-glucosidase were determined to be 941.01 ± 21.45, 4.46 ± 0.17, and 6.26 ± 0.07 μg/mL. Results showed that both LLP-D and LLP-W exhibited extremely stronger inhibitor effects against α-glucosidase than that of acarbose, indicating that LLPs had the potential applications to prevent and manage diabetes.

In general, natural polysaccharides can inhibit enzymatic activities of α-amylase and α-glucosidase through binding to the enzymes and changing their structures [22,23]. Polysaccharides with low molecular weight are easy to bind with the active sites of enzymes [31,33]. Besides, acidic polysaccharides can increase the binding capacity due to the presence of electrophilic groups, such as keto or aldehyde [22]. Therefore, the low molecular weight and high content of uronic acids of LLP-D may contribute to its strong inhibitory activities against α-amylase and α-glucosidase. Besides, the high viscosity and high degree of esterification of LLP-D could also benefit its hypoglycemic activity [34]. Overall, LLP-D extracted by DES could be served as a new and natural hypoglycemic substance in the functional food industry.

### 3.5. Immunomodulatory Effects of LLPs

**Figure 8:**
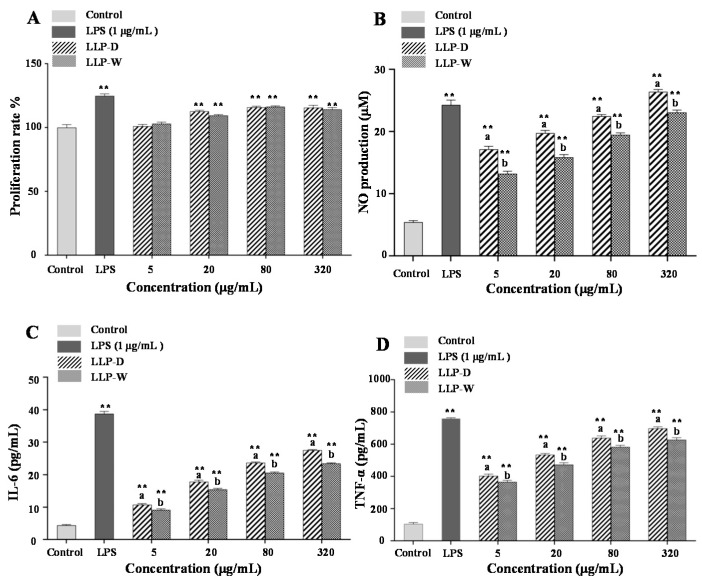
Effects of LLP-D and LLP-W on the cytotoxicity (**A**), NO production (**B**), IL-6 production (**C**), and TNF-α production (**D**) of RAW 264.7 macrophages. LLP-D and LLP-W are polysaccharides from lotus leaves extracted by deep eutectic solvent and hot water, respectively; Each experiment was operated in triplicate, and the error bars are standard deviations; Significant differences of cell viability and productions of NO, IL-6, and TNF-α of LLP-W and LLP-D vs. control are shown by ** *p* < 0.01. Superscripts a-b differ significantly (*p* < 0.05) between LLP-W and LLP-D.

Several studies have reported that polysaccharides extracted from lotus leaves can activate RAW 264.7 macrophages [5,7]. Therefore, in this study, the LPS induced-RAW 264.7 macrophage was used as a model for the evaluation of immunomodulatory effects of LLP-W and LLP-D. Figure 8 shows the effects of LLP-W and LLP-D on the cytotoxicity, NO, IL-6, and TNF-α productions of RAW 264.7 macrophages. As shown in Figure 8A, both LLP-W and LLP-D showed no toxicity effects on RAW 264.7 macrophages. Moreover, results showed that both LLP-W and LLP-D notably promoted the production of NO, IL-6, and TNF-α in a dose-dependent manner, respectively. At the concentration of 320 μg/mL, the productions of NO, IL-6, and TNF-α from macrophages activated by LLP-W and LLP-D ranged from 23.08 ± 0.40 to 26.40 ± 0.38 μM, from 23.44 ± 0.21 to 27.51 ± 0.20 pg/mL, and from 627.46 ± 12.48 to 698.52 pg/mL, respectively. Notably, compared with LLP-W, LLP-D exhibited much stronger immunomodulatory effects, suggesting that the DESE method also possessed good potential for preparation of acidic polysaccharides with high immunomodulatory effects from lotus leaves.

Generally, the immunomodulatory effects of natural polysaccharides are correlated to the structural characters, such as molecular weight, monosaccharide composition, degree of esterification [7,35]. Previous studies have shown that the low molecular weight polysaccharides often exhibit strong immunomodulatory effects [7,36,37]. Besides, the immunomodulatory effects of acidic polysaccharides are positively correlated to the content of uronic acids and the degree of esterification [7,35,38]. Therefore, compared with LLP-W, the stronger immunomodulatory effects of LLP-D might be attributed to its relatively lower molecular weight, higher content of uronic acids, and higher degree of esterification (Table 3). However, in order to reveal the detailed structure-function relationships of LLP-D, the further purification, structural characterization, and in vitro and in vivo bioactivities evaluation are required in the future.

## 4. Conclusions

In this study, the maximum extraction yield (5.38%) of LLPs was obtained under the optimal extraction conditions as 61.0% of water content in DES, extraction temperature of 92.0 °C, liquid-solid ratio of 31.0 mL/g, and extraction time of 126.0 min. Compared with hot water extraction (yield, 3.22%), DES was more efficient for extracting polysaccharides from lotus leaves. Furthermore, LLP-D and LLP-W possessed the same constituent monosaccharides and glycosidic linkages. Indeed, the main polysaccharide fractions, including HG, AG, and RG I, could be found in both LLP-W and LLP-D. Notably, the DESE method could be beneficial for specific extraction of HG fraction from lotus leaves. Furthermore, compared with LLP-W, LLP-D exhibited lower molecular weight and higher content of uronic acids (GalA), which might contribute to its stronger in vitro antioxidant, hypoglycemic, and immunomodulatory effects. Hence, these results suggest that the DESE method can be a potential method for specific extraction of acidic polysaccharides with high bioactivities from lotus leaves.

## Figures and Tables

**Table 1:** The Box-Behnken design with independent variables and observed values of the extraction yield of polysaccharides from lotus leaves.

Runs	Levels of Independent Parameters				Yields (%)
	X_1_ (%)	X_2_ (°C)	X_3_ (mL/g)	X_4_ (min)	
1	0 (60)	−1 (80)	0 (30)	1 (150)	3.20
2	0 (60)	1 (100)	−1 (20)	0 (120)	4.01
3	1 (50)	−1 (80)	0 (30)	0 (120)	2.41
4	1 (70)	0 (90)	1 (40)	0 (120)	4.06
5	1 (50)	1 (100)	0 (30)	0 (120)	3.51
6	−1 (50)	0 (90)	−1 (20)	0 (120)	3.10
7	0 (60)	0 (90)	0 (30)	0 (120)	5.40
8	0 (60)	1 (100)	0 (30)	1 (150)	4.47
9	0 (60)	0 (90)	−1 (20)	1 (150)	4.06
10	0 (60)	−1 (80)	0 (30)	−1 (90)	2.96
11	1 (70)	0 (90)	0 (30)	−1 (90)	3.27
12	0 (60)	0 (90)	0 (30)	0 (120)	5.27
13	−1 (50)	0 (90)	1 (40)	0 (120)	3.35
14	0 (60)	0 (90)	0 (30)	0 (120)	5.32
15	0 (60)	1 (100)	1 (40)	0 (120)	4.31
16	0 (60)	−1 (80)	−1 (20)	0 (120)	2.97
17	−1 (50)	0 (90)	0 (30)	−1 (90)	3.01
18	1 (70)	−1 (80)	0 (30)	0 (120)	3.20
19	0 (60)	0 (90)	1 (40)	−1 (90)	3.84
20	0 (60)	0 (90)	0 (30)	0 (120)	5.38
21	0 (60)	0 (90)	0 (30)	0 (120)	5.42
22	−1 (50)	0 (90)	0 (30)	1 (150)	3.52
23	0 (60)	0 (90)	−1 (20)	−1 (90)	3.46
24	0 (60)	−1 (80)	1 (40)	0 (120)	3.38
25	1 (70)	0 (90)	0 (30)	1 (150)	3.76
26	0 (60)	1 (100)	0 (30)	−1 (90)	3.41
27	1 (70)	0 (90)	−1 (20)	0 (120)	3.39
28	0 (60)	0 (90)	1 (40)	1 (150)	4.17
29	1 (70)	1 (100)	0 (30)	0 (120)	3.35

X_1_, content of water in DES; X_2_, extraction temperature; X_3_, liquid-solid ratio; X_4_, extraction time.

**Table 2:** Analysis of the variance for the fitted second-order polynomial model for deep eutectic solvent assisted extraction.

	Sum of Squares	df	Mean Square	*F*-Value	*p*-Value
Model	19.51	14	1.39	147.7	<0.0001 **
X_1_	0.44	1	0.44	47.13	<0.0001 **
X_2_	2.18	1	2.18	231.54	<0.0001 **
X_3_	0.37	1	0.37	39.70	<0.0001 **
X_4_	0.87	1	0.87	92.15	<0.0001 **
X_1_X_2_	0.15	1	0.15	15.71	0.0014 **
X_1_X_3_	0.044	1	0.044	4.67	0.0484 *
X_1_X_4_	1.000 × 10^−4^	1	1.000 × 10^−4^	0.011	0.9195
X_2_ X_3_	3.025 × 10^−3^	1	3.025 × 10^−3^	0.32	0.5802
X_2_X_4_	0.17	1	0.17	17.82	0.0009 **
X_3_X_4_	0.018	1	0.018	1.93	0.1863
X_1_^2^	9.03	1	9.03	957.01	<0.0001 **
X_2_^2^	6.80	1	6.80	720.31	<0.0001 **
X_3_^2^	3.01	1	3.01	318.91	<0.0001 **
X_4_^2^	4.18	1	4.18	442.57	<0.0001 **
Residual	0.13	14	9.435 × 10^−3^		
Lack of fit	0.12	10	0.012	3.06	0.1464
Pure error	0.1464	4	3.820 × 10^−3^		
Correlation total	19.64	28			

R^2^ = 0.9933, R^2^_adj_ = 0.9866, coefficient of variation (CV) = 2.53%, and adeq. precision = 43.16; X_1_, content of water in DES (%); X_2,_ extraction temperature (°C); X_3_, liquid-solid ratio (mL/g); X_4_, extraction time (min); Significantly different, * *p* < 0.05, ** *p* < 0.01.

**Table 3:** Chemical compositions, molecular weight (*M_w_*), and molar ratios of constituent monosaccharides of polysaccharides extracted from lotus leaves.

	LLP-W	LLP-D
**Yields and chemical compositions**		
Extraction yield (%)	3.22 ± 0.16 ^b^	5.38 ± 0.11 ^a^
Total polysaccharides (%)	77.94 ± 0.48 ^b^	82.10 ± 1.29 ^a^
Total uronic acids (%)	22.98 ± 1.15 ^b^	39.96 ± 0.32 ^a^
Total proteins (%)	3.39 ± 0.39 ^b^	5.97 ± 0.52 ^a^
Degree of esterification (%)	16.46 ± 0.08 ^b^	28.92 ± 0.77 ^a^
***M_w_*** **× 10^4^ (Da)**		
Peak 1 (15 min to 20 min)	12.90 ± 0.09	-
Peak 2 (20 min to 21 min)	4.70 ± 0.07	-
Peak 3 (21 min to 22 min)	3.43 ± 0.09	-
Peak 4 (17 min to 21 min)	-	4.03 ± 0.07
**Monosaccharides and molar ratios**		
Galactose	1.00	1.00
Galacturonic acid	0.70	1.36
Arabinose	0.64	0.70
Rhamnose	0.21	0.27
Mannose	0.26	0.10
Glucose	0.22	0.19
Glucuronic acid	0.21	0.18
Xylose	trace	trace

LLP-D and LLP-W are polysaccharides from lotus leaves extracted by deep eutectic solvent and hot water, respectively; Each experiment was operated in triplicate, and values represent mean ± standard deviation. Superscripts a and b differ significantly (*p* < 0.05) between LLP-W and LLP-D.

## Data Availability

Not applicable.
